# Variables correlated with elderly referral from nursing homes to general hospitals

**DOI:** 10.1186/2045-4015-3-2

**Published:** 2014-01-23

**Authors:** Shir Wagman, Shmuel Rishpon, Genady Kagan, Jonathan Dubnov, Sonia Habib

**Affiliations:** 1 Ministry of Health, Jerusalem, Israel; 2 Haifa District Health Office, Ministry of Health, Haifa, Israel; 3 Faculty of Welfare and Health Sciences, School of Public Health, University of Haifa, Haifa, Israel

**Keywords:** Elderly, Hospitalization, Nursing homes, Referral rates

## Abstract

**Background:**

Referring patients from nursing homes to general hospitals exposes them to nosocomial diseases, and may result in the development of a broad spectrum of physical, mental and social damages. Therefore, minimizing the referring of nursing home patients to hospitals is an important factor for keeping the elderly healthy and minimizing health expenditures. In this study we examined the variables related to the referral rates from nursing homes to general hospitals and the relationship between the referral and the mortality rates among the elderly who live in nursing homes in the Haifa Sub-district.

**Methods:**

Thirty-two nursing homes were included in a cross-sectional study. All medical directors and head nurses were interviewed using a structured questionnaire between November 2006 and October 2007. Statistical analysis, including the ANOVA and the nonparametric Spearman tests, were employed to determine the factors that influence referral rates and the correlation between referral rates and mortality rates.

**Results:**

The referral rate ranged between 18 and 222 per 100 financed elderly in a single year. In the multivariate analysis, the absence of a physician from the nursing home at the time of the referral to general hospitals was the only significant variable related to referral rates. No significant relationships were found between referral rates and mortality rates.

**Conclusions:**

Absence of a significant relationship between referral rates and mortality rates may indicate that high referral rates do not necessarily protect the elderly from death. Therefore, any recommendations issued by the Ministry of Health (MOH) should emphasize in-house treatment rather than hospitalization. Clear instructions on referral from nursing homes to general hospitals need to be constructed by the MOH. The MOH should increase the presence of physicians in the nursing homes, especially, when the need to refer a patient arises. Further quantitative and epidemiologic studies should be conducted in order to, more fully and reliably, create guidelines for policy recommendations.

## Background

Nursing homes (NHs) are characterized by nursing and supportive treatment for elderly patients suffering from cognitive disorders and patients who need constant, prolonged attention and care [1]. While general hospitals are characterized by medical expertise and medical technologies, there is minimal focus on prolonged treatment. In hospitals, the level of nursing and patient care for the elderly is lower than it is in NHs. The referral of patients from NHs to general hospitals often exposes them to iatrogenic diseases and can result in psychological and social damage [2]. Thus, minimizing hospitalizations is important for keeping the elderly healthy as well as for limiting costs [2]. Socio-demographic characteristics, health characteristics, nursing staff related characteristics and the availability of other services are variables that affect the referring of elderly patients to general hospitals [3]. Hospitalization in a general hospital causes a decline in the nutritional status of the patient and additional, often infectious, complications. Among patients suffering from malnutrition, higher rates of repeated hospitalizations and higher mortality rates were observed. For elderly patients, hospitalization in itself is a factor that increases the risk of functional decline by 35% during the hospital stay [1,4]. Severe respiratory infections, fractures and pressure wounds are other common complications reported among the elderly as a result of hospitalization [5,6]. Additionally, the transfer to the hospital, a new and unknown environment, creates an inconvenience for the elderly patient [7].

Acute respiratory infections are the most common causes for referring elderly patients from nursing homes to hospitals [8]. The above factors contribute to a higher mortality rate and increase the chance for other severe consequences, like pressure wounds, among the hospitalized, when compared with non-hospitalized elderly patients who are taken care of at the nursing home [4].

Nursing home related variables, like private ownership, belonging to a network of NHs, poor environmental conditions, a lack of patient privacy and poor satisfaction among the elderly and staff, are related to higher rates of elderly patient referral from NHs to hospitals [9,10].

In a study conducted by Menec (2008), patient related variables like younger age, general deterioration and a need for additional assistance in Activities of Daily Living (A.D.L.) contribute to higher rates of hospitalizations. In this study 41% of elderly patients were hospitalized at least once in general hospitals during the six months before they died [11].

The decision of nursing home staff to refer patients to a general hospital is difficult. Lacking clear instructions from the Ministry of Health, the staff might feel obligated to refer patients to hospitals despite potentially severe consequences [7].

About half of elderly patients’ referrals from NHs to hospitals are not justified and occur regardless of preliminary instructions [12].

Medical managers and head nurses have different perceptions with respect to the factors that affect patients’ referral to general hospitals. Commonly perceived factors include the availability of physicians, their payment level and the preferences of the patients’ families. These differences in perception might impose difficulties on conducting effective interventions directed at minimizing referral rates. Improving communication and reaching a consensus among the staff regarding patient referrals to general hospitals are important components. Furthermore, preliminary instructions and treatment according to the patients’ needs will minimize referral rates [13].

Elderly patients in NHs receive 24 hour availability of nursing staff and caregivers, as well as the presence of medical and paramedical staff. However, there is still a widespread phenomenon of referring elderly patients in NHs to general hospitals for medical care. Therefore, it is important to identify the factors correlated with elderly referral from NHs to general hospitals. In this study, we examined the factors related to elderly referral rates from NHs to general hospitals in the Haifa sub-district, as well as the correlation between referral rates and mortality rates in these NHs, from November 2006 to October 2007, in order to determine the needs for an intervention policy to reduce the referring of elderly patients to general hospitals. Such a study has not been conducted yet in Israel.

## Methods

The Haifa sub-district has a total population of approximately 550,000 residents, 16% of whom are above 64 years old. Thirty-three licensed NHs which contain a total of about 2,500 beds are located within the Haifa sub-district. Thirty-two of the NHs are connected to the Haifa District Health Office by the SAP^®^ (Systems, Applications, and Products in Data Processing) software. The medical managers and the head nurses (senior staff) of the above mentioned 32 NHs were interviewed using a structured questionnaire that included the following variables: [1] qualification and availability of staff in the NHs; [2] referring professionals; and [3] knowledge and attitude of senior staff members on the topic of elderly referral.

Data about the NHs was collected using the SAP^®^ software in the Haifa District Health Office, (HDHO). Collection of data occurred between 1/11/2006 and 31/10/2007 and included several variables such as the number of licensed beds in the NHs, the number of elderly referrals to general hospitals for each NH, the number of hospital admissions, the duration of the admission in days, and the total number of deaths for each NH. The data referred only to elderlies whose NH stay was financed by the Ministry of Health, (MOH), (locally known as “codes”), as computerized data was readily available for this group. Later in the study, the NHs were divided into two groups: larger NHs with greater than 50 licensed beds, and smaller NHs with fewer than 50 licensed beds. This was done in order to examine the size of a NH as an independent variable that may influence the referral rate. The senior staff of each NH was interviewed between May and August 2009. They were asked to consider the average referral rate in their NH in comparison with the average referral rate in the Haifa sub-district. The referral rate, which was the main dependent variable, was defined as the number of patient referrals divided by the total number of financed elderly throughout a single year. We examined the relationship between the referral rate and the independent variables: the medical managers’ availability (hours per week), medical managers’ specialty, geriatric nurse training, nurses’ qualification, referral form details (whether directly by the physician or indirectly through the nurse), NH size, and the professional knowledge of the senior staff. The professional knowledge of the senior staff was examined using four theoretical scenarios about the medical status of elderly patients. The mortality rate was defined as the number of deaths divided by the total number of financed elderly throughout a single year.

Statistical analysis, using SPSS software, included univariate and multivariate analysis. Referral rates were investigated using variance analysis (ANOVA) to determine whether the mean referral values, observed in different groups of NHs, differ by the above-mentioned categorical variables. The Spearman nonparametric correlation test was performed to determine the correlation between the mortality rate and the referral rate. A value of P < 0.05 was considered statistically significant.

## Results

The study population included 32 NHs out of the 33 licensed NHs in the Haifa sub-district. These NHs constituted a convenient sample due to their connection with SAP^®^ software and available computerized data. One NH was not included due to a lack of connection with this software. Out of the 32 NHs, 17 were considered big and 15 were considered small. The referral rate distribution and main characteristics of NHs are demonstrated in Table 1.

**Table 1:** **Characteristics of Nursing Homes (NHs) by number of beds category (small/large) in Haifa sub district in 2006**–**2007 (standard deviation and percentages in parenthesis)**

	Small NHs* **N** = **15**	Large NHs** **N** = **17**	**P value**
Specialized medical manager (%)	6 (40.0%)	7 (41.2%)	.615
Physician availability per day			
Up to 8 hours	10 (66.7%)	5 (29.4%)	.102
9-12 hours	2 (13.3%)	6 (35.3%)	
13 and more hours	3 (20.0%)	6 (35.3%)	
Registered nurse availability per shift (%)			
Morning	4 (26.7%)	2 (11.8%)	.550
Morning and afternoon	4 (26.7%)	6 (35.3%)	
Morning, afternoon and night	7 (46.7%)	9 (52.9%)	
Referral form (%)			
Direct	10 (66.7%)	15 (88.2%)	.149
Indirect	5 (33.3%)	2 (11.8%)	
Average referral rate per 100 financed beds (SD)***	69.1 (SD = 54.0)	63.7 (SD = 28.1)	.722
Average annual mortality rate per 100 financed beds (SD)	35.3 (SD = 19.2)	31.3 (SD = 15.8)	.525
Average length of stay in days in general hospitals (SD)	7.2 (SD = 3.3)	6.4 (SD = 1.5)	.400

Note: *Small *NHs* (<=49 licensed beds): were included 506 beds with 162 referrals to general hospitals in study period. **Large *NHs* (> = 50 licensed beds): were included 1829 beds with 887 referrals to general hospitals in study period. ***Financed by Ministry of Health.

The availability of the medical managers ranged from 9 to 12 hours per week in fifteen of the NHs (47%), 24 hours per week in six of the NHs (19%) and one hour per week in one NH. Nineteen (59%) of the medical managers were general practitioners and seven physicians were specialists in geriatrics. In 18 NHs (56%), more than 50% of the nurses were registered nurses. In 19 NHs (59%) qualified geriatric-trained nurses were employed. In all NHs, a registered nurse was available during the morning shift, in 81.3% of the NHs, a registered nurse was available during the afternoon shift, and in 50% of the NHs, a registered nurse was available during the night shift. Only seven NHs have permission to conduct intravenous treatment. The senior staff of 17 of the NHs had a success rate of 75% or more in the knowledge exam (had a correct answer for at least 3 out of the 4 knowledge-oriented medical scenarios).

The referral rate ranged between 18 and 222 per 100 financed elderly in a single year, with a median of 60. A wide variation of referral rates was found among NHs. No statistical difference was detected between the referral rates of big and small NHs, (Chi^2^ = 0.08, P value = 0.783). Therefore, further analysis was performed for all NHs.

In twenty-five NHs (78%) the physician was available, examined the patient, and referred him directly. Conversely, in seven NHs (22%) the physician was not available and the nurse consulted with him by phone. The referral in this case was done indirectly by sending a referral form by fax.

Lower referral rates were observed when direct referrals were made by physician than when indirect referrals were made by a registered nurse. This difference was significant, F =7.461, p ≤ 0.01 (Table 2).

**Table 2:** **Referral rates distribution according to characteristics of Nursing Homes in Haifa sub district in 2006**–**2007 (univariant ANOVA procedure)**

**Variable**	**N**	**Average referral rate**	**F (df)**	**P value**
Specialized medical manager:				
General	19	73.9	1.641	0.210
Specialized	13	55	(1)	
Physician availability per day				
Up to 8 hours	15	75.7	0.787	0.465
9–12 hours	8	61.6	(2)	
13 and more hours	9	54.6		
Registered nurse availability per shift				
Morning	6	72.3	0.106	0.900
Morning and afternoon	10	62.2	(2)	
Morning, afternoon and night	16	66.5		
Referral form*				
Direct	25	56.6	7.461	0.010
Indirect	7	100.7	(1)	
Knowledge among medical managers				
No correct answers	1	98		0.854
One answer of the four correct	4	82.7	0.333	
Two answers of the four correct	9	61.1	(4)	
Three answers of the four correct	15	63.9		
All answers correct	3	60.7		

*Significant.

Lower referral rates were also observed to be dependent upon the physicians’ specialization and availability. However this relationship was not significant, F = 1.641, p = 0.210 and F = 0.787, p = 0.465, respectively.

Analysis of variance (ANOVA) showed no significant relationship between referral rates and the other variables including physician availability and specialty, registered nurse availability and senior staff knowledge (Table 2).

In multivariate analysis, the referral form variable remained significant and demonstrated the lowest rates in case of direct referral to general hospitals by physicians (t = -2.128, P = 0.043).

Multivariate analysis showed no significant relationship between referral rates and the other variables, including senior staff knowledge, physician availability and specialty, and nurse availability, qualification and training.

Eighty-one percent of the medical managers in NHs where the referral rates were lower than the median reported that the referral rates of their NH, in their opinion, were low. On the other hand, 44% of the medical managers in NHs where the referral rates were higher than the median reported that the referral rates of their NH, in their opinion, were high. The relationship between the attitude of medical managers and the referral rates in their NH’s was borderline significant, Chi^2^ = 4.886, P value = 0.087. No significant relationship between referral rates and mortality rates was found, Pearson Correlation = -0.025, P value = 0.892.

## Conclusions

In this study we examined the relationship between elderly referral rates and a mix of human resources, staff knowledge and other characteristics of NHs. The study population included 32 NHs out of the 33 licensed NHs in the Haifa sub-district. These NHs constituted a convenient sample due to their connection with SAP^®^ software and available computerized data. One NH was not included due to a lack of connection with this software. The data, included in this study, referred only to elderlies whose NH stay was financed by the Ministry of Health, (MOH), (locally known as “codes”), as computerized data was readily available for this group. Including data about elderly patients whose hospitalization was not financed by the MOH, proved difficult as the data, relevant to them, was not computerized. This is a good topic to be included in a future study.

A significant decrease in referral rates was observed when the physician was present in the NHs and referred the patient directly. In seven NHs, the physician was not available and the nurse consulted with him by phone. The patient referral in this case was done indirectly by sending a referral form by fax. In these NHs, higher referral rates were observed. Lower referral rates were also observed to be dependent upon the physicians’ specialization and availability. However this relationship was not significant. Higher referral rates may be attributed to the fact that some NHs did not offer malpractice insurance for employed physicians. This, in turn, may have led the physicians to prefer referring patients to a general hospital to taking responsibility for an in-house treatment. This hypothesis was not examined in this study. Since 2008, NHs listed by the MOH as approved for financed hospitalization after going through the bidding process, offer general insurance to all employed physicians.

In Israel, NHs are paid for 2 weeks after elderly referral and hospitalization in a general hospital, even though they are allowed to admit another elderly patient during this time, and to be paid for them as well. This might render the decision to refer a patient to a general hospital fairly “easy”, considering the potential profit of such an action. This hypothesis was not examined in this study.

No significant relationship was found between the referral rates and the percentage of qualified nurses, geriatric training among nurses and permission to conduct intravenous treatment in the NHs. The non-significant relationship demonstrated between the referral rates and those variables may be attributed to the small sample size of NHs included in the study.

In published studies, referral rates are influenced by the characteristics of NHs and the quality of service they provide. [3,9]. Availability of qualified nurses in nursing homes was found to be directly related to elderly referral rates and due to health conditions like diabetes mellitus, COPD, dehydration, pneumonia and urinary tract infections [14]. Increased nursing staff skills and interventions were related to a decrease in referral rates [2]. Availability of qualified nurses, permission to conduct intravenous treatments, and training interventions among medical and nursing staff were related to a decrease in referral rates [10,14]. Lack of qualified nurses in NHs was related to higher referral rates [15]. Permission to conduct intravenous treatment in NHs has led to a decrease in elderly referral rates by 40%-50% [5].

In our study no significant relationship was found between referral rates and mortality rates. This finding may suggest that higher referral rates do not necessarily lead to higher mortality rates nor do they protect the patients from death.

### Limitations

1. Our study is population-based and included a convenient small sample of all the NHs in the Haifa sub-district (32 NHs). This might explain the absence of a significant relationship between referral rates and most of the independent variables. Moreover, because of the small sample size, the findings may be biased. Expansion of the sample size of the NHs is required for future studies.

2. No data regarding the medical causes for elderly referral was collected.

3. The data about mortality rates was population-based. No individual follow up of a hospitalized patient was conducted. Further individual study is required in order to determine the relationship between referral rates and mortality rates.

### Recommendations

1. An additional study, including data about the elderlies whose NH stay is not financed by the MOH, should be considered.

2. Further quantitative and epidemiologic studies should be conducted in order more fully and reliably create guidelines for policy recommendations.

3. The MOH should issue criteria for the referral of patients from a nursing home to a general hospital.

4. MOH should increase the presence of physicians in the NHs, especially when the need to refer a patient arises. MOH should require the physician to be present in cases where a referral is to be issued.

5. MOH should consider the option of encouraging referrals to geriatric emergency rooms.

6. Similar studies should be conducted among other NHs in other districts. Further studies are required to evaluate the intervention.

## Competing interests

The authors declare that they have no competing interests.

## Authors’ contribution

SW and GK initiated the study and took the lead in its planning and implementation. SH took the lead in writing the article. All of the authors (SH, SW, GK, JD and SR) participated in the study’s planning and implementation, as well as the preparation of the manuscript. All of the authors have reviewed and approved the final manuscript.

## Author’s information

*Sonia Habib* is a public health specialist, Deputy District Health Officer in Haifa Health Office and acting Head of Haifa Sub District health office and a teacher at the Public Health School, Haifa University. *Shir Wagman* is Deputy Head Nurse, Public Health Services, Ministry of Health. *Genady Kagan* is the District Geratrician, in Haifa District of Health Office. *Jonathan Dubnov* is a public health specialist, Deputy District Health Officer in Haifa Health Officeand a lecturer at the Public Health School, Haifa University. *Shmuel Rishpon* is a public health specialist, Haifa District Health Officer, an Associate professor at the Public Health School, Haifa University and Head of the Advisory Committee on Infectious Diseases and Immunizations.
